# P10-03 Developing online health community platform for enhancing physical activity in the community

**DOI:** 10.1093/eurpub/ckac095.142

**Published:** 2022-08-29

**Authors:** Myonghwa Park, Jihye Jung, Jahyeon Kim, Jinju Kim

**Affiliations:** College of Nursing, Chungnam National University, Daejeon, Republic of Korea

**Keywords:** online, health, platform, physical activity, community

## Abstract

Virtual communities are emerging in many aspects of health activities and widespread in health management. Online health communities offer a virtual system where people with common interest, specific health needs can exchange information and experiences with other people with the same condition as well as getting support from peer and professionals. The objective of this study is to develop an ICT platform enhancing community resident participation involving in the chronic disease prevention and physical activities. The aim of this platform is to promote physical activity of community residents for health promotion and disease prevention goal attainment. Health promotion goals for enhancing physical activity were set by health care professionals based on scientific evidence. Instead of individual plan to set the goal by themselves, the platform offered them tailored goal with their conditions and then suggested them to participate in group shared their physical activity goals. The platform encouraged the active participation of community residents though adherence physical activity of health communities in which they were members.The ICT platform provides a place where community residents with chronic conditions or even healthy people who want to promote their health be able to find an appropriate group for together prevent disease and enhancing physical activity. The platform in the study allowed community residents to develop their own communities and invite other members to participate with them. This online community intended to empower community residents to increase their involvement in their self-management and pushing the active participation in phycial activities. Function such as reminder of activity participating was added. Future study will be conducted to evaluate changes in health promotion self-efficacy, health goal fulfillment, health-related quality of life, and shared decision-making after using the Health Community Platform among community residents. Extensive research initiatives are needed to determine the impact of virtual health communities on patient outcome, the overall process as well as quality and access of care.

